# Treatment of Unresectable Differentiated Thyroid Carcinoma With Upfront External Radiotherapy and Salvage Surgery: A STROBE-Compliant Retrospective Cohort Study

**DOI:** 10.3389/fonc.2020.572958

**Published:** 2021-01-19

**Authors:** José F. Carrillo, Jesús Manuel Flores, Gilberto Espinoza, Rafael Vázquez-Romo, Margarita C. Ramírez-Ortega, Liliana C. Carrillo, Beatriz Y. Cortés-García, Francisco J. Ochoa-Carrillo, Luis F. Oñate-Ocaña

**Affiliations:** ^1^ Departamento de Cabeza y Cuello, Instituto Nacional de Cancerología (INCan), Mexico City, Mexico; ^2^ Departamento de Radioterapia, Instituto Nacional de Cancerología (INCan), Mexico City, Mexico; ^3^ Subdirección de Cirugía, Instituto Nacional de Cancerología (INCan), Mexico City, Mexico; ^4^ Direccion de Investigación, Instituto Nacional de Cardiología Ignacio Chávez, Mexico City, Mexico; ^5^ Subdirección de Investigación Clínica, Instituto Nacional de Cancerología (INCan), Mexico City, Mexico; ^6^ Departamento de Imagen, Instituto Nacional de Cancerología (INCan), Mexico City, Mexico

**Keywords:** thyroid neoplasms, radiotherapy, thyroid surgery, locally advanced neoplasms, salvage surgery

## Abstract

**Introduction:**

In patients with unresectable Differentiated thyroid cancer (DTC), the use of external beam radiation therapy (EBRT), leads mostly to palliation. Our aim is to define the role of upfront EBRT, followed or not by salvage surgery, on Progression-free survival (PFS) or Overall survival (OS) in patients with DTC.

**Methods:**

This is a cohort study of patients with initially unresectable DTC who received EBRT. Cohort A received EBRT followed by rescue surgery and cohort B, EBRT only. The Kaplan-Meier method and Cox model were employed for survival analysis.

**Results:**

Thirty-three patients were included; 69.6% females and 30.3% males. Mean age was 60.6 and mean tumor diameter was 10.4 cm; 17 and 16 patients were included in cohorts A and B, respectively. Belonging to cohort A (Hazard ratio [HR] 0.177, 95% CI 0.05–0.7) and use of intensity modulated radiotherapy (HR 0.177, 95% CI 0.03–1.08) were associated to better PFS, while high-risk histopathology (HR 6.6, 95% CI 0.9–50) and EBRT dose (HR 1.05, 95% CI 1.01–1.08) were independently associated with lower PFS. Patients from cohort A (HR 0.061, 95% CI 0.01–0.3) had improved OS, while high-risk histopathology (HR 5.7, 95% CI 1.1–28.6) and EBRT dose (HR 1.05, 95% CI 1.01–1.09) were independently associated to worse OS.

**Conclusion:**

EBRT, and when feasible, salvage surgery, should be an integral part of the therapeutic strategy in initially unresectable DTC.

## Highlights

- Is unresectable differentiated thyroid cancer due to vascular invasion responsive to external beam radiotherapy, and if feasible, salvage surgery improves the oncologic outcome?
- In this cohort study, 17 patients received external beam radiation therapy followed by salvage surgery, and 16 received external beam radiation therapy only. Radiation therapy produced a high response rate which allowed successful salvage surgery, with increased disease-free and overall survival.
- Radiation therapy, and when feasible, salvage surgery should be considered in these patients; as this is a retrospective cohort study and subject to selection bias, a randomized trial is warranted.

## Introduction

Thyroid cancer incidence is rising worldwide (1, 2). In Mexico, it is the second malignant neoplasm in women (3). Differentiated thyroid cancer (DTC) is mostly treated with total thyroidectomy and subsequent ^131^I radioiodine to ablate residual thyroid tissue or micrometastases. However, finding unresectable DTC is infrequent and not significantly addressed (2, 4).

The indications for external beam radiotherapy (EBRT) have been reported previously. The use of EBRT in patients with high-risk features for relapse and gross residual disease has been described and subject to controversy (5). Unresectable DTC, patients have been treated with EBRT, mainly to obtain palliation (5, 6).

Series on unresectable DTC analyze patients who have received upfront, non-operative treatment, with EBRT, some including chemotherapy and target-aimed agents, but clinical responses or subsequent surgical resection are not described, aside from anecdotal cases (7–9). Moreover, current American Thyroid Association (ATA) guidelines do not clearly define the role of EBRT in cases with advanced unresectable DTC (10), although other guidelines suggest a palliative role of EBRT with improvement in quality of life and local disease control (11, 12).

Radioiodine resistant, locorregionally unresectable, or recurrent DTC have been treated with tyrosine kinase inhibitors like Lenvatinib. Although reports are encouraging, only 36% of patients have a partial response with high toxicity rates and increased costs (13).

Therefore, our aim is to describe the results of upfront EBRT in patients with initially unresectable DTC, and when feasible, of the addition of surgery.

## Materials and Methods

### Patients

A retrospective cohort study of patients with unresectable, locally-advanced DTC admitted at our institution between January 2006 and December 2016. Inclusion criteria were patients older than 17, histopathological confirmation, normal serum thyroid function tests, no previous treatment, and the presence of unresectable neoplasms. Clinical history and physical exam, naso-pharyngo-laryngoscopy, Computed tomography (CT), and Magnetic resonance angiogram of the neck and chest areas were obtained in all cases. High risk histopathology meant a poorly DTC defined by five or more mitoses per 10 high‐power microscopic fields, lack of nuclear features of papillary thyroid carcinoma, and/or the presence of tumor necrosis; besides, the presence of aggressive variants of papillary thyroid carcinoma, like insular, tall cell, sclerosing, columnar, or hobnail variants were included in this category as well (14). Unresectable DTC was defined as T4a/T4b DTC and/or metastatic lymph nodes with invasion and more than 180° –without cleavage plane– surrounding of the following vascular structures: vascular structures of the root of the neck (innominate veins and artery, as well as subclavian-carotid systems), superior vena cava and/or pulmonary artery system, anterosuperior mediastinum, as well as extension to spine and neurovascular structures –including vertebral artery–, which precluded a complete excision of the mass without major risk for function or life (9).

Patients with bone and visceral metastases, except for cases with six or fewer nodular lung metastases and no evidence of pleural effusions, were excluded.

Tumor recurrence and locoregional progression were defined as the appearance of new tumor lesions or an increase in size of existent primary lesions in the neck or superior mediastinum, after R0 surgical resection or after radiotherapy completion, respectively, demonstrated by biopsy or cytology, and/or imaging findings.

Distant metastases were defined as the appearance of new lesions outside the neck and upper mediastinum, detected clinically or with imaging studies.

This study protocol is STROBE-compliant, and was accepted by “Comité de Ética en Investigación” and “Comité de Investigación” (registration number Rev/0002/2019). Written consent was not required because of the retrospective nature of the study.

### External Beam Radiation Therapy

All patients received upfront EBRT. Before 2012, 3D conformal EBRT technique was used; subsequently, intensity modulated radiation therapy (IMRT).

The standard EBRT fractionation protocol and doses above 56 Gy were used if patients had an ECOG ≤ 2. Otherwise, a hypo-fractionation palliative course of 30–50 Gy was used. For 3D conformal technique, volume delineated in all patients was the Gross tumor volume (GTV); with doses of 56–80 Gy for patients with low ECOG. The clinical target volume (CTV) included the primary tumor and a five mm margin around the GTV: the neoplastic thyroid gland volume, tracheoesophageal grooves, central nodal compartment, and all positive nodal levels were delineated in the simulation and planning by CT.

In patients with poor ECOG, the palliative doses never exceeded 45 Gy in the GTV; the most frequently used doses were 23 and 30 Gy in 10 fractions.

IMRT and methods to integrate dose levels according to risk of recurrence were implemented according to the specialist criteria. Regions considered low risk for microscopic disease were treated with a 46 Gy dose. The ‘‘low-risk’’ clinical target volume (CTV) included lymph node-negative areas in the cervical neck, and the upper mediastinum to the level of the carina.

Patients were immobilized and CT images obtained every 3 mm from the skull vertex to the lungs. IMRT was planned with a gradient inverse algorithm with dose-volume constraints as previously defined (5, 6), using the Eclipse™ software and the Varian IX™ accelerator (Varian Medical Systems, Inc., Palo Alto, Ca).

Response was assessed according to the RECIST criteria (15), and all measurements were performed on angio-CT scans by one CT-interpretation specialized radiologist.

### Surgical Technique

Cohort A included candidates for surgical resection because of a partial RECIST response and/or with sufficient separation of the major blood vessels, which allowed surgical resection after EBRT. Cohort B included patients who refused surgery, or that were still considered unresectable after EBRT because of stable or progressive disease and consequently, surgical resection was not considered.

Surgery consisted of a total thyroidectomy with unilateral or bilateral neck dissection, in accordance with the affected lymph nodes. If needed, an anterior mediastinotomy with innominate venous and arterial system dissection was performed. Central node dissection was conducted in all cases as well. Tracheal resection was performed when indicated, as well as a postoperative tracheostomy.

### Follow-Up

After treatment, patients were followed with thyroid function tests and thyroglobulin levels at three-month intervals, and CT and/or MRI every 6 months. Thyroid suppression was administered to all patients during and after EBRT administration and after surgical resection.

### Statistical Analysis

Student’s-t or Chi-square tests were used as required. Prognostic factors analyzed were age, gender, tumor diameter, histopathology, T, N, and M classifications, EBRT dose, protraction, dose per fraction, EBRT technique, ^131^I therapy, ^131^I dose, and clinical response according to RECIST criteria. Survival data were analyzed with the Kaplan-Meier and Logrank methods and the Cox’s model. PFS was calculated from EBRT initiation to recurrence/progression event; OS was calculated from diagnosis until the last visit or death. Prognostic factors associated with PFS or OS with a probability value of 0.2 or less, by bivariate analysis, were included in the multivariate analyses. Hazard ratios (HR) were obtained as a measurement of association with 95% confidence intervals (95% CI). The final model was selected according to accomplishment criteria and goodness-of-fit tests; the final model was examined for proportionality assumptions and interaction terms.

Neither final exposure variables nor outcome data presented missing values. Missing data analysis showed that missingness were at random; multivariate regression imputation was employed as required. Probabilities of 0.05 or less were considered significant, two-tailed statistics were used, and SPSS statistical software for Mac, version 23 (IBM Corp., Armonk, NY, USA) were employed.

## Results

### Patients

Of 1,438 patients with DTC, 33 (2.29%) met the inclusion criteria; 23 (69.6%) were females and 10 were males (30.3%). Mean age was 60.6 years (Standard deviation [SD] 14.6). Mean tumor diameter was 10.35 cm (SD 4.2). Complete resection after EBRT was achieved in 17 cases (cohort A); 16 cases were allocated in cohort B. Fewer patients in cohort B received ^131^I radioiodine treatment, fewer received EBRT by IMRT technique and fewer had response by RECIST than patients in cohort A (**Table 1**).

**Table 1:** Demographic and clinical characteristics of patients by cohort (N=33).

Factor		Cohort A (*N*=17)	Cohort B (*N*=16)	*P*
Gender*	Female	12 (70.5)	11(68.7)	0.603
	Male	5 (31.2)	5 (29.4)	
**Age**	(years)	54.9 (13.1)	66.6 (14)	0.019
**Tumor diameter**	(cm)	8.70 (3.8)	12 (4.6)	0.031
Histopathology*	classic	15 (88.2)	14(87.5)	0.948
	high-risk	2 (11.8)	2(12.5)	
T classification*	T4a	2(11.7)	2(12.5)	0.909
	T4b	15(88.2)	14(87.5)	
N classification*	N1b	17(100)	16(100)	0.948
M classification*	M0	14 (82.4)	10 (62.5)	0.198
	M1	3 (17.6)	6 (37.5)	
**EBRT dose**	(Gy)	51.4 (20.6)	53.9 (18.9)	0.708
**Protraction**	(weeks)	12.01 (16.5)	11.3 (14.8)	0.894
**Dose per fraction**	(Gy)	3.12 (1.96)	2.95 (1.61)	0.777
EBRT technique*	ARC	9 (52.9)	14 (87.5)	0.027
	IMRT	8 (47.1)	2 (12.5)	
^131^I therapy*	(received)	16 (94.1)	3 (18.8)	<0.0001
**^131^I Dose**	(mCi)	223.3 (75.3)	183.3 (28.9)	0.387
Clinical Response*	(RECIST)			
Progressive or stable disease		0	3 (18.8)	0.061
Partial response		17 (100)	13 (81.3)	

N, number of patients; *categorical variables and numbers are absolute counts (percentages); others are continuous variables and numbers represent means (standard deviation); EBRT, external beam radiotherapy; ARC, conformational; IMRT, intensity modulated; Gy, Grays; mCi, milliCuries; P, probability values.

### EBRT

Eight (47.1%) and two (12.5%) patients in cohorts A and B received IMRT respectively, while nine (52.9%) and 14 (87.5%) cases had conformational therapy, respectively. Patients in cohort A received a mean dose of 51.4 Gy (SD 20.6) and those in cohort B 53.9 Gy (SD 18.9). Other treatment characteristics with radiation therapy by cohorts are listed in demographic and descriptive **Tables 1** and **2**. All patients in cohort A had partial responses (PR) or a response which permitted surgery. In cohort B three cases had progressive or stable disease (18.8%), other three patients (18.8%) a partial response, and the rest a minor response insufficient to qualify for PR according to RECIST criteria. No case in our series reached a complete response.

**Table 2:** Description of cases of differentiated thyroid carcinoma treated with upfront radiotherapy and surgery.

No.	Age	G	TD	T	N	M	EBRT Technique	Dose (Gy)	Sx	R	ECOG	P	RAI Dose	Status
1	83	M	8	T4b	N1b	0	Conformal	69	Yes	R0	1	WD	300	DWD
2	53	F	8	T4b	N1b	0	Conformal	65	Yes	R0	1	WD	300	DWD
3	67	F	8	T4a	N1b	0	Conformal	65	No		1	WD	0	DWD
4	68	F	10	T4b	N1b	0	Conformal	70	No		1	tall cells	0	DWD
5	67	F	12	T4b	N1b	0	Conformal	65	No		1	PD	0	DWD
6	75	M	8	T4a	N1b	1	Conformal	45	No		2	WD	150	DWD
7	74	F	10	T4b	N1b	1	Conformal	70	No		1	WD	0	DWD
8	55	F	10	T4b	N1b	0	Conformal	30	No		3	WD	0	DWD
9	64	F	7	T4a	N1b	1	Conformal	66	Yes	R0	1	WD	350	DWD
10	54	M	5	T4a	N1b	0	Conformal	30	Yes	R0	3	WD	150	AWOD
11	30	F	8	T4b	N1b	0	Conformal	40	Yes	R0	1	WD	150	AWOD
12	54	F	6	T4b	N1b	0	IMRT	45	Yes	R0	1	PD	200	AWOD
13	60	M	12	T4b	N1b	1	IMRT	66	Yes	R0	1	WD	200	AWOD
14	43	F	8	T4b	N1b	0	Conformal	23	Yes	R0	3	WD	200	AWOD
15	47	F	8	T4b	N1b	1	Conformal	23	Yes	R0	3	WD	200	AWD
16	61	F	5	T4b	N1b	0	Conformal	43	Yes	R0	2	WD	200	AWOD
17	50	F	20	T4b	N1b	0	IMRT	60.2	Yes	R2-0	1	WD	200	AWOD
18	78	F	8	T4b	N1b	0	Conformal	70.2	No		1	WD	200	DWD
19	82	F	8	T4b	N1b	0	IMRT	60.2	Yes	R0	1	WD	150	DWD
20	34	F	15	T4b	N1b	0	IMRT	70	No		1	WD	0	AWD
21	46	F	7	T4b	N1b	0	IMRT	68	Yes	R0	1	WD	150	AWOD
22	83	F	18	T4b	N1b	1	Conformal	47	No		1	WD	0	DWD
23	75	F	18	T4b	N1b	0	Conformal	45	No		2	WD	0	DWD
24	48	F	7	T4b	N1b	0	Conformal	43	Yes	R0	2	WD	250	AWOD
25	77	M	15	T4b	N1b	0	Conformal	30	No		3	WD	0	AWD
26	38	M	20	T4b	N1b	0	Conformal	23	No		3	WD	0	AWD
27	48	M	8	T4b	N1b	0	IMRT	46	Yes	R0	2	Insular	0	DWOD
28	67	M	18	T4b	N1b	1	Conformal	23	No		3	WD	0	AWD
29	58	F	8	T4b	N1b	1	Conformal	74	No		1	WD	0	DWD
30	50	F	15	T4b	N1b	0	IMRT	66	Yes	R2	1	WD	250	AWOD
31	71	F	6	T4b	N1b	1	IMRT	66.6	No		2	WD	200	DWD
32	79	M	8	T4b	N1b	0	Conformal	70	No		2	WD	0	DWD
33	61	M	8	T4b	N1b	0	IMRT	60	Yes	R0	1	WD	100	AWOD

No., number; G, Gender; TD, Tumor Diameter; EBRT, External Beam Radiotherapy; IMRT, Intensity Modulated Radiotherapy; Gy, Grays; Sx, Surgery; R, Resection type; P, Pathology; WD, Well differentiated; RAI, Radioactive Iodine; AWOD, Alive Without Disease; AWD, Alive with disease; DWD, Dead With Disease; DWOD, Dead without Disease.

Radiation therapy-associated morbidity included grade III radio-epithelitis and xerostomia in seven (21%) and eight cases (24.4%), respectively. Dysphagia due to mucositis occurred in 12 (36%) cases and required percutaneous endoscopic gastrostomy (PEG). In four cases (12.12%) in which tracheal invasion was present, upper airway obstruction led to performance of a tracheostomy. One of these patients has not been decannulated after 4 years of follow-up, while the rest are now deceased.

Initially, there were three (9.09%) cases with lung metastases. Metastases developed during follow-up in nine cases (27.27%), and included five lung (15.5%), two bone (6.06%) and two brain metastases (6.06%).

### Surgery

The median interval time between EBRT and surgery was 13.27 months (2.3–81). Total thyroidectomy and unilateral neck dissection were performed in 14 cases, with bilateral neck dissection in three. Additional mediastinotomy was performed in three cases, and tracheal resection in two. One patient who had surgery had an R2 resection, and was subjected to a second surgical procedure which included mediastinotomy and reached an R0 status. The rest of the cohort A patients had an R0 status after surgery. Postoperative morbidity included temporal and permanent laryngeal nerve injury in five (29.4%) and five cases (29.4%), respectively. Transient and permanent hypoparathyroidism occurred in one (5.8%) and two (11.6%) cases, respectively. There was major surgical bleeding (≥500 ml) in five (29.4%) cases, pneumothorax in four (23.5%), and a chylous fistula in four (23.5%).

There were two cases each with high- risk histopathology in Cohorts A and B.

Three patients (9.09%) had no surgery based on a personal decision, although they were candidates for surgical resection and were included in cohort B. One of them had progression at 14 months follow-up, and the other two are alive and with no signs of locoregional progression or metastases.

There was one case of surgery-related death due to laceration of the innominate artery which was repaired by carotid artery-proximal innominate artery Gore-Tex grafting and right subclavian artery ligation. Patient developed a major stroke during the following 12-h period and pronounced dead and was included as an event in the survival analysis.

From cohort A, three patients had locoregional recurrences which were treated with neck dissection. Patients developed progressive lung metastases and died in spite of treatment with ^131^I radioiodine and TSH supression. Another case had progression of already present lung metastases which were treated with ^131^I radioyodine, TSH suppression, and sorafenib with no response. Finally, a fifth patient had lung metastases progression treated with ^131^I radioyodine, TSH suppression and is alive and stable with lung disease.

### Survival

Median follow-up was 2.74 years (0.5–7.9). Median PFS in all patients was 3.72 years (95% CI 2.2–5.2). There were 17 recurrence/progression events in both cohorts (51.5%); five were in cohort A (29.4%) and 12 in cohort B (75%) (*p* = 0.009). Median PFS in cohorts A and B were 6.03 (95% CI 3.37–8.7) and 0.89 years (95% CI 0–3.82), respectively.

Age, presence of distant metastases, ^131^I radioiodine therapy administration, EBRT dose, and patients who underwent surgery (cohort A) were significantly associated with PFS by bivariate analysis (**Table 3**). High-risk histopathology, Conformational EBRT technique, EBRT total dose, and absence of surgery (cohort B) were independent explanatory factors associated with lower PFS by multivariate analysis (**Table 4**).

**Table 3:** Bivariate association of various factors and progression-free or overall survival (N = 33).

Factor		HR (95% CI) PFS	*P (PFS)*	HR (95% CI) OS	*P(OS)*
Gender*	Female	1	0.449	1	0.487
	Male	0.214 (0.13–0.1.59)		0.669 (0.22–2.076)	
**Age**	(years)	1.038 (1.003–1.07)	0.032	1.038 (1.002–1.08)	0.036
**Tumor diameter**	(cm)	1.013 (0.89–1.14)	0.831	1.013 (0.9–1.14)	0.829
Histopathology*	classic	1	0.968	1	0.586
	high-risk	0.968 (0.2–4.59)		1.446 (0.383–5.45)	
T classification*	T4a	1	0.446	1	0.74
	T4b	0.61 (0.17–2.18)		0.802 (0.22–2.95)	
M classification*	M0	1	0.011	1	0.167
	M1	4.221 (1.39–12.9)		2.072 (0.737–5.82)	
**EBRT dose**	(Gy)	1.033 (1.002–1.06)	0.034	1.037 (1.004–1.07)	0.029
**Protraction**	(weeks)	0.998 (0.97–1.03)	0.923	0.998 (0.966–1.03)	0.901
**Dose per fraction**	(Gy)	0.746 (0.49–1.12)	0.156	0.587 (0.33–1.054)	0.075
EBRT technique*	ARC	1	0.088	1	0.218
	IMRT	0.274 (0.06–1.21)		0.454 (0.13–1.594)	
^131^I therapy*	(received)	0.309 (0.1–0.89)	0.029	0.205 (0.06–0.66)	0.008
**^131^I Dose**	(mCi)	1.002 (0.99–1.01)	0.67	1 (0.991–1.009)	0.987
Clinical Response*	(RECIST)				
Progression or stable disease		1	1	1	0.0002
Partial response		1 (0.009–112.1)		0.032 (0.005–0.2)	
**Cohort**	B	1	0.003	1	0.003
	A	0.144 (0.4–0.52)		0.102 (0.02–0.46)	

N, number of patients; *categorical variables; HR, Hazard ratios; CI, confidence intervals; PFS, progression-free survival; OS, overall surviva;T: tumor classification, M: metastases classification; EBRT, external beam radiotherapy; ARC, conformational; IMRT, intensity modulated; Gy, Grays; mCi, milliCuries; P, probability value.

**Table 4:** Multivariate association of various factors and progression-free survival or overall survival (*N* = 33).

Factors (outcome PFS)		β	S.E.	expβ (HR)	(95% CI)	*P*
**High-risk histopathology**		1.9	1.031	6.688	0.89–50.5	0.065
**EBRT technique**	(IMRT)	–1.731	0.926	0.177	0.029–1.08	0.062
**EBRT dose**	(Gy)	0.045	0.016	1.046	1.01–1.08	0.005
**Cohort**	B	–	–	1	–	– 0.013
	A	–1.731	0.7	0.177	0.045–0.69	
**Factors (outcome OS)**						
**High-risk histopathology**		1.738	0.825	5.686	1.129–28.6	0.035
**EBRT dose**	(Gy)	0.051	0.019	1.053	1.014–1.093	0.007
**Cohort**	B	–	–	1	–	– 0.001
	A	–2.803	0.843	0.061	0.01–0.317	

N, number of patients; β, regression coefficient; S.E., standard error of the regression coefficient; expβ, beta exponent or HR, Hazard ratios; CI, confidence intervals; P, probability value; PFS, progression-free survival; OS, overall survival; EBRT, external beam radiotherapy; Gy Grays (PFS model -2 Log Likelihood = 64.1; p=0.000082; OS model -2 Log Likelihood 66.6; p = 0.000017).

Median OS of all patients was 5.07 years (95% CI 3.1–6.9). There were 17 events of death in both cohorts (51.5%); five in cohort A (29.4%), and 12 in cohort B (75%) (Logrank *p*=0.009). Median OS in cohorts A and B was 6.9 (95% CI 5.5–8.2) and 1.6 (95% CI 0–4.7) years, respectively.

Age, ^131^I radioiodine therapy administration, EBRT dose, clinical response by RECIST and inclusion in cohort A were associated with OS by bivariate analysis (**Table 3**). High-risk histopathology, EBRT dose, and belonging to cohort B were independent explanatory factors associated with worse OS by multivariate analysis (**Table 4**).

**Figure 1:**
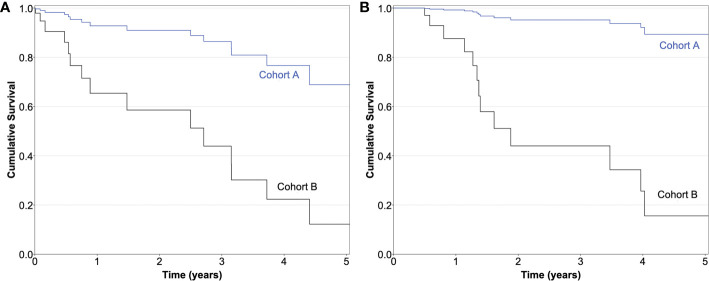
**(A)** Progression-free survival curves of both cohorts constructed using the final Cox model (adjusted for high-risk histopathology, presence of metastases, and total external beam radiotherapy dose as shown in **Table 3**; model *p* = 0.000017). **(B)** Overall survival curves of both cohorts constructed using the final Cox model (adjusted for high-risk histopathology and total external beam radiotherapy dose as shown in **Table 3**; model *p* = 0.000082).

The proportionality assumptions were satisfactory in both models. The presence of interaction terms was investigated in both final models and none were found. **Figure 1** depicts the PFS and OS survival curves by cohort (constructed using the final Cox models described in **Table 4**).

### Representative Case

**Figure 2:**
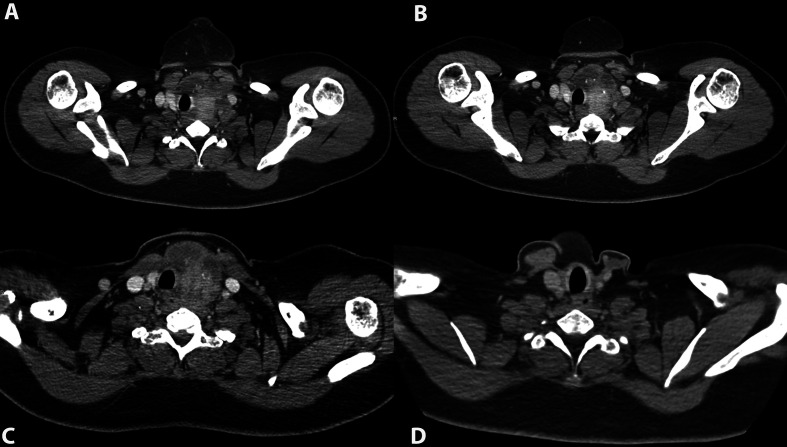
**(A)** Contrast computed tomography (CT) where a primary neoplasm of the left thyroid lobe and isthmus extends laterally to the common carotid artery with >180° encasement of same vessel and extension to prevertebral fascia, where no cleavage plane is observed. This lesion is associated to lymph node metastases in level III which encases partially the internal jugular vein (IJV) and produces major compression of the esophagus. **(B)** Lower axial view of the aforementioned neoplasm where extent to the mediastinum is demonstrated as well. Encasement of the left carotid artery, and major compression of the esophagus are demonstrated. **(C)** Contrast CT after EBRT (external beam radiotherapy treatment) where decrease in size of the aforementioned lesion located in the left thyroid lobe is observed. At this time, no major encasement of the carotid artery exists and a cleavage plane with common carotid artery and prevertebral fascia is demonstrated. Increase in soft tissue density is related to previous radiotherapy. The esophagus and IJV are non-compressed by the malignancy. **(D)** Contrast CT after a total thyroidectomy, left lateral, and central neck dissection. No suspicious lesions are observed, trachea and esophagus have no morphology alterations.

**Figure 2** shows the case of a 38-year-old female, diagnosed with DTC who received IMRT radiotherapy in 2014, with more than 30% response. She underwent a total thyroidectomy and left modified radical neck dissection in 2016, and is currently disease-free.

## Discussion

Patients with locally advanced DTC are treated with surgery, which sometimes includes extensive procedures; some researchers considering radiation therapy as complementary, and in R0 cases, as adjuvant treatment (5, 6).

When the extent of malignancy is associated with invasion to vascular structures of the base of the neck and upper mediastinum precluding a safe and clear resection, the therapeutic options are limited to observation, symptomatic treatment, and/or palliative EBRT.

Some isolated case-series exist where DTC cases considered unresectable receive chemotherapy, targeted agent therapy or EBRT and then subjected to a surgical procedure, occasionally attaining complete resection (7, 8). The objective of these anecdotal reports is to underscore palliation, with an R0 resection obtained in one or two cases.

A 20% and 50% three-year locoregional control has been obtained in patients treated with palliative or radical radiotherapy, respectively, in unresectable DTC, with no further attempt to perform surgery after EBRT. Frequently, cases in these analyses had undergone incomplete surgical resections (16–18).

Current guidelines consider EBRT administration indicated in cases where surgery has been performed but with gross residual disease, and even in these circumstances, radiotherapy use is controversial (10, 12).

Partial resection and complementary radiotherapy are less efficient because of the presence of scarred, neoplastic and non-oxygenated tissue after treatment. Further, the possibilities of obtaining a cure are nil.

Our patients were considered unresectable due to major invasion of the vascular structures of the base of the neck and upper mediastinum, including the innominate vein and artery systems, as well as the subclavian-carotid and aortic arch structures.

Main findings of our study are that EBRT has an efficient role in management of unresectable DTC and that IMRT plus salvage surgery were associated with an increased PFS/OS, while high-risk histopathology decreased PFS (**Table 3**).

In this study, the median PFS in patients who underwent salvage surgery was six years, and was superior to the findings of Terezakis (5), who described that patients left with gross residual disease had locoregional control (LRC) of 62% at 4 years, with no attempt to perform surgery after EBRT. The improvement in PFS associated with the use of IMRT has been suggested in previous reports (19), where no surgery was attempted. Although the number of cases treated with IMRT was small (ten cases), partial response occurred in eight (80%) which allowed an R0 resection, while surgical resection with conformal radiotherapy was achieved in 39% of cases.

Higher doses of EBRT were associated to decreased DFS and OS by multivariate analyses, probably because of selection bias resulting from need to treat larger tumors more aggressively.

As expected, the presence of aggressive histopathology was confirmed to be a factor associated with an increased risk of recurrence, progression and death.

In this cohort study, with a median follow-up of 2.74 years, successful rescue surgery was independently associated with improved PFS and OS. This finding underscores the significance of a complete resection when feasible, since these patients usually receive a palliative treatment.

A word of caution should be given, since our objective was not a comparison between EBRT treated cases and those who received Surgery plus EBRT, but to underline a positive effect of EBRT on unresectable DTC, with better oncologic outcomes when surgery becomes feasible.

This is the first report, to our knowledge, in which a comprehensive evaluation and a salvage surgical resection are attempted in initially unresectable DTC in patients who previously received an upfront EBRT course.

The time interval before surgical intervention was performed in operable cases was a median of 13.3 months, which underscores the long-term effect of radiotherapy on DTC, which has not been previously described. Aside, the potential deleterious effect of fibrosis caused by EBRT ─with the increased risks for a surgical procedure─ is diminished after this time period has elapsed.

We devised a different strategy of not performing debulking surgery, as previously reported, but to administer a radical dose of EBRT. Subsequently, surgical feasibility was evaluated with the potential advantage of preventing tumor dissemination and scarring after an R2 procedure which in turn, could preclude surgical rescue after radiotherapy administration (20).

Metastases were infrequent in these cohorts, of not major significance on PFS or OS, specifically in the multivariate analyses; this suggests that our strategy has potential impact in the control of this factor since it allows the administration of ^131^I radioiodine with improvement in the oncologic outcome.

EBRT toxicity was significant; the most frequent complications were upper airway and esophageal obstruction, which were managed with temporary tracheostomy and PEG. In spite of the extensive radiation fields, no major or death-related toxicity was reported in our series. Although there was a trend toward a greater incidence of toxicity with conformal therapy, this had no significance; lower toxicity rates are obtained with the administration of IMRT (21), and secondarily, a greater target-directed dose delivery.

In our cohorts, aside from greater frequencies of hypoparathyroidism and laryngeal nerve palsy, either transitory or permanent, than those reported, there was a significant percentage of cases with major bleeding and pneumothorax, and one patient died during the procedure due to laceration of the innominate artery. In spite of this relatively high rate of surgical complications, these are expected to occur due to the extent of dissection and the presence of scarring and edema in previously radiated tissues (20).

Same consideration could be applied to the one death reported (where even with a partial response and complete dissection of the lesion) infiltration of the great vessels persisted and led to uncontrollable bleeding. Even after a major response to EBRT, we believe that if the lesion is still encircling the great vessels by more than 180°, and there is dubious invasion or contact with pulmonary vessels, surgery should not be attempted, although there are case reports of venous and arterial grafting, albeit with a high operative death rate (20), early recurrence and the development of metastases.

In spite of this drawback, radiotherapy in our patients – especially after the administration of IMRT– contributed to the creation of a lower tumor volume and more defined dissection planes, thus allowing a safer and realistic surgical plan to conduct an R0 resection in initially non-resectable cases.

The retrospective nature of our data and relatively small number of cases, are the main drawbacks of this study. However, and given the paucity of unresectable DTC cases who are candidates for upfront EBRT and eventual rescue surgery, this cohort study constitutes, to our knowledge, one of the largest available.

Other limitations include the change over time from conformational 3D therapy to IMRT, and the lack of a health-related quality of life evaluation. Although the change in EBRT technique increased the heterogeneity of our analyses, it also allowed us to conduct an internal comparison of the efficiency of both methods, resulting in a clear advantage of IMRT in terms of PFS. HRQL was not formally evaluated in this cohort, but the decreased complication rate associated to IMRT and the oncologic outcomes obtained after resection suggest that, in future multicenter studies, the application of the strategy described will lead to better PFS and OS results, and consequently HRQL.

Ideally, a randomized controlled trial would be the instrument required to settle the controversy on the role of EBRT and potential salvage surgery in patients with initially unresectable DTC. However, previous attempts to perform an adequate randomized controlled trial, have been closed early due to the small numbers of recruited patients (21).

## Conclusion

A favorable LRC rate and OS after EBRT in unresectable DTC cases secondary to major invasion of the mediastinum and lower neck vascular structures is demonstrated. Moreover, EBRT results improved significantly with the addition of surgery.

EBRT [especially IMRT (20, 22)] and when feasible, salvage surgery, should be an integral part of the management of unresectable DTC.

## Data Availability Statement

The original contributions presented in the study are included in the article/supplementary material. Further inquiries can be directed to the corresponding author.

## Ethics Statement

The studies involving human participants were reviewed and approved by Comité de Investigación and Comité de Etica en Investigación. Written informed consent for participation was not required for this study in accordance with the national legislation and the institutional requirements.

## Author Contributions

Study concepts: JC, JF, FO-C, and LO-O. Study design: JC, RV-R, and LO-O. Data acquisition: JF, GE, RV-R, LC, and BC-G. Quality control of data and algorithms: JC, GE, BC-G, FO-C, and LO-O. Data analysis and interpretation: JC, MR-O, LC, FO-C, and LO-O. Statistical analysis: JC, MR-O, LC, and LO-O. Manuscript preparation: JC, RV-R, BC-G, FO-C, and LO-O. Manuscript editing: JC, JF, GE, MR-O, LC, FO-C, and LO-O. Manuscript review: All authors. All authors contributed to the article and approved the submitted version.

## Conflict of Interest

The authors declare that the research was conducted in the absence of any commercial or financial relationships that could be construed as a potential conflict of interest.
